# Supplementary material for: Strong ethanol- and frequency-dependent ecological interactions in a community of wine-fermenting yeasts
Source: Commun Biol. 2023 Sep 13;6:939. doi: 10.1038/s42003-023-05284-1 (PMC10499898; doi:10.1038/s42003-023-05284-1)
Supplement: Supplementary file 2 — Supplementary Information [file 42003_2023_5284_MOESM2_ESM.docx]

**Supplementary Figure 1: Growth rates in a synthetic grape juice like medium are barely affected by removing up to 75% of the carbon or nitrogen sources in the medium.** We measured the growth of each of our five species in synthetic grape juice, where we independently varied the concentration of carbon and nitrogen resources relative to their standard concentrations. In almost all cases, removal of up to 75% of the resource had negligible effects on growth, highlighting how growth dynamics in the early stages of fermentation, when resources remain abundant, are not primarily driven by decreasing resource concentration.

**Supplementary Figure 2 (next page): Consensus interaction networks average the outcomes of three experimental replicates.** The blue stars in some Rep II networks indicate that the two initial conditions of the same pair did not reach the same outcome, with one condition reaching exclusion and one maintaining coexistence (at the fraction indicated by the blue star) at the end of the 7-day cycle. The gray arrows in the Rep II 5ABV network indicate that the coculture collapsed.


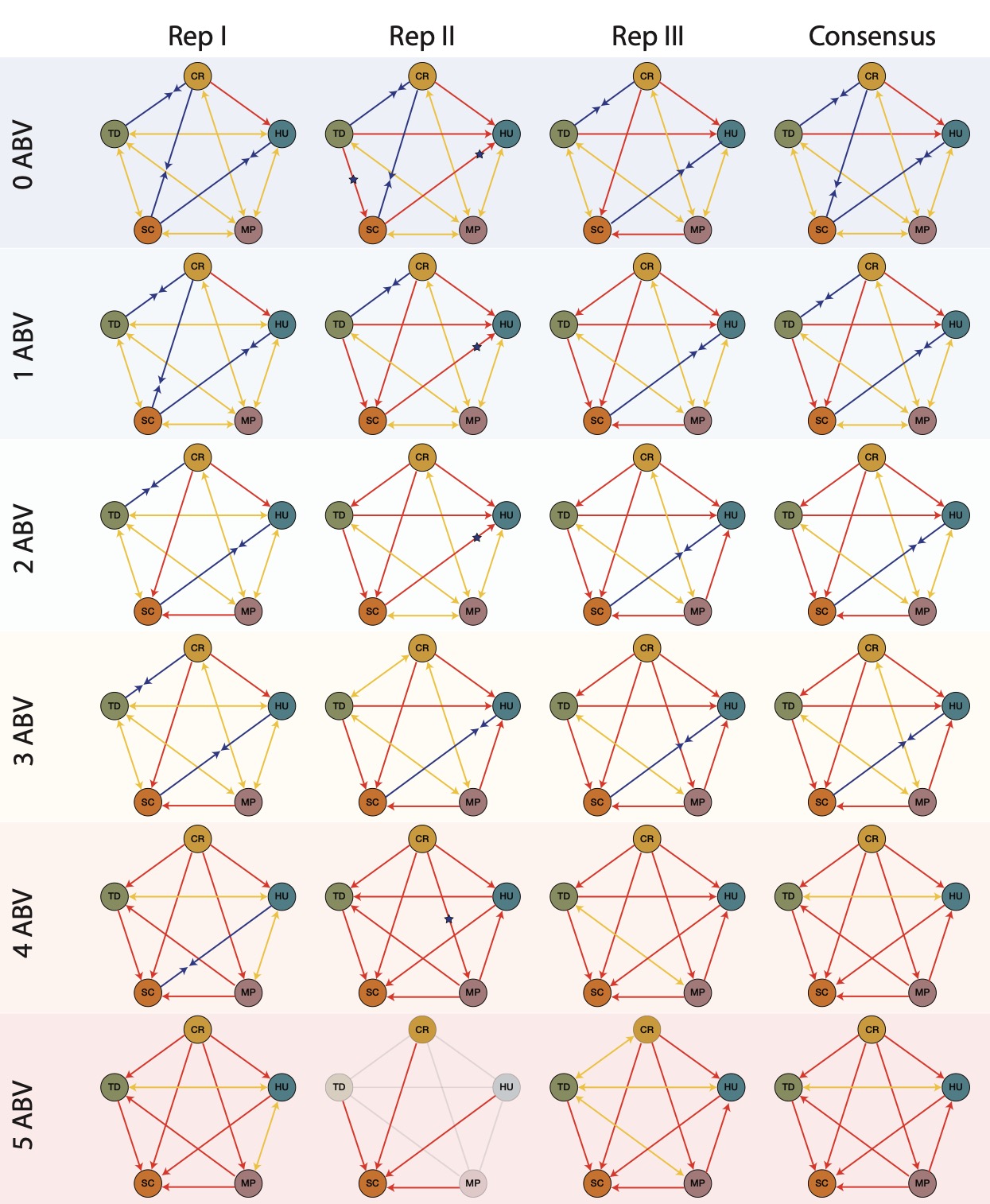


|  |  |  |  |  |  |  |
| --- | --- | --- | --- | --- | --- | --- |
|  |  | **Pairwise Competitive Score** | | | |  |
|  |  | **Growth Rate** | | **K** | |  |
| ***ABV*** |  | *Corr* | *p* | *Corr* | *p* |  |
| ***0*** |  | 0.691 | 0.196 | -0.048 | 0.939 |  |
| ***1*** |  | 0.533 | 0.355 | 0.243 | 0.693 |  |
| ***2*** |  | 0.186 | 0.765 | 0.478 | 0.415 |  |
| ***3*** |  | 0.272 | 0.657 | 0.486 | 0.406 |  |
| ***4*** |  | 0.383 | 0.525 | 0.88 | 0.049 |  |
| ***5*** |  | 0.884 | 0.047 | 0.739 | 0.153 |  |
|  |  |  |  |  |  |  |
|  |  | **5 Species Competitive Score** | | | |  |
|  |  | **Growth Rate** | | **K** | |  |
| ***ABV*** |  | *Corr* | *p* | *Corr* | *p* |  |
| ***0*** |  | 0.66 | 0.225 | -0.008 | 0.99 |  |
| ***1*** |  | -0.161 | 0.796 | 0.833 | 0.08 |  |
| ***2*** |  | 0.419 | 0.482 | 0.173 | 0.781 |  |
| ***3*** |  | -0.072 | 0.908 | 0.57 | 0.315 |  |
| ***4*** |  | 0.498 | 0.393 | 0.484 | 0.408 |  |
| ***5*** |  | 0.595 | 0.29 | 0.571 | 0.315 |  |
|  |  |  |  |  |  |  |
|  |  |  |  |  |  |  |
|  |  | **Pairwise vs. 5 SP** | |  |  |  |
| ***ABV*** |  | *Corr* | *p* |  |  |  |
| ***0*** |  | 0.988 | 0.002 |  |  |  |
| ***1*** |  | 0.644 | 0.241 |  |  |  |
| ***2*** |  | 0.908 | 0.033 |  |  |  |
| ***3*** |  | 0.668 | 0.218 |  |  |  |
| ***4*** |  | 0.806 | 0.099 |  |  |  |
| ***5*** |  | 0.892 | 0.042 |  |  |  |
|  |  |  |  |  |  |  |

**Supplementary Table 1: Pearson correlations and their associated p-values for comparisons between (Top) two-species competitive outcomes and growth rates and carrying capacity (K), (Center) five-species competitive outcomes and growth rates and carrying capacity (K), and (Bottom) two-species competitive outcomes and five-species competitive outcomes.**
